# A GSH Fluorescent Probe with a Large Stokes Shift and Its Application in Living Cells

**DOI:** 10.3390/s19245348

**Published:** 2019-12-04

**Authors:** Yueyuan Mao, Yediao Xu, Zhi Li, Yang Wang, Huanhuan Du, Lei Liu, Ran Ding, Guodong Liu

**Affiliations:** 1 College of Chemistry and Materials Engineering, Anhui Science and Technology University, Bengbu 233030, China; 15656052160@139.com (Y.X.); lizhiusta@163.com (Z.L.); 18855036950@163.com (Y.W.); dhh122808@163.com (H.D.); liulei@feipan.org (L.L.); dingran128@126.com (R.D.); 2 State Key Laboratory of Molecular Engineering of Polymers, Fudan University, Shanghai 200433, China; 3 Institute of Biomedical and Health Science, Anhui Science and Technology University, Bengbu 233030, China

**Keywords:** fluorescent probe, GSH, cell imaging

## Abstract

Intracellular GSH is the most abundant non-protein biothiol and acts as a central antioxidant to defend against aging toxins and radicals. Meanwhile abnormal level of intracellular GSH concentration is directly related to some diseases. In this case, detecting intracellular GSH rapidly and sensitively is of great significance. We synthesize a simple fluorescent probe (named **GP**) which can discriminate GSH from Cys (cysteine) or Hcy (homocysteine) and presents a 50-fold fluorescence increasing. The response time of **GP** to GSH was only 5 min and the product **GO** (the product of **GP** after reacting with GSH) after reacting with GSH possesses a larger Stokes shift for 135 nm than that in reported work. Probe **GP** can detect intracellular effectively and shows obvious yellow fluorescence. Briefly, probe **GP** can detect intracellular GSH rapidly and effectively both in vitro and in living cells.

## 1. Introduction

Biothiols containing glutathione (GSH), cysteine (Cys) and homocysteine (Hcy) play significant roles in maintaining the normal redox status in biological systems [1,2,3]. However, GSH, a tripeptide which is made up of glutamic acid, cysteine, and glycine [4], is the most abundant non-protein biothiol and acts as a central antioxidant to defend against aging toxins and radicals [5,6]. Sulfhydryl reduced form (GSH) and disulfide oxidized form (GSSG) are two existing forms in vivo and they maintain the redox homeostasis via the transformation between GSH and GSSG [7]. The concentration of GSH ranges from micromole in saliva, plasma, and other body fluids to millimole in cells [8]. Meanwhile, an abnormal level of GSH concentration is directly related to some diseases, such as retarded children, hepatic impairment, and even serious diseases such as Alzheimer’s disease [9], cardiovascular diseases [10], diabetes mellitus [11], cancer, etc. As the structure of GSH was extremely similar to that of Cys or Hcy, it is a tough mission to discriminate GSH from Cys and Hcy.

Familiar methods to detect GSH mainly include electrochemistry [12], colorimetry [13], surface-enhanced Raman scattering [14], magnetic resonance imaging [15], mass spectrometry [16], electrochemical luminescence [17], and fluorescence spectroscopy [18]. Among those detection methods, fluorescent analysis is a much simpler, more efficient and lower destructive method and detecting limits, which can be applied in living cells and even in vivo to imaging bio-thiols. In this case, development of a stable, sensitive, efficient and fast responsive fluorescent probe to GSH is meaningful both in vitro and in vivo.

Fluorescent probes can be classified into two categories: inorganic materials and organic molecules. Inorganic materials applied to detect GSH mainly take advantage of the reduction properties of GSH [19]. Most organic fluorescent probes which were applied to detect GSH showed high nucleophilicity, which were named “reaction based probes”. Reaction based probes are mainly related to cleavage of sulfonate ester [20], acryloyl ester [7] and aryl substitution reactions [5,21,22], disulfide bond cleavage [23] and cyclization [24], and Michael additions [25,26]. As organic molecules are easy to modify, fluorescent probes with different emission wavelengths were synthesized and applied to cell imaging. Benzothiazoles derivatives have rigid planar structure, high fluorescence quantum yield, low toxicity, etc., which have important applications in photoelectric materials [27] and fluorescent probes [28,29,30,31]. The reported benzothiazole-based derivatives mainly emit blue or green light, which is limited for application in cell imaging. Therefore, it is of great significance to develop benzothiazole-based probes with longer fluorescence emission wavelength.

**Scheme 1:**
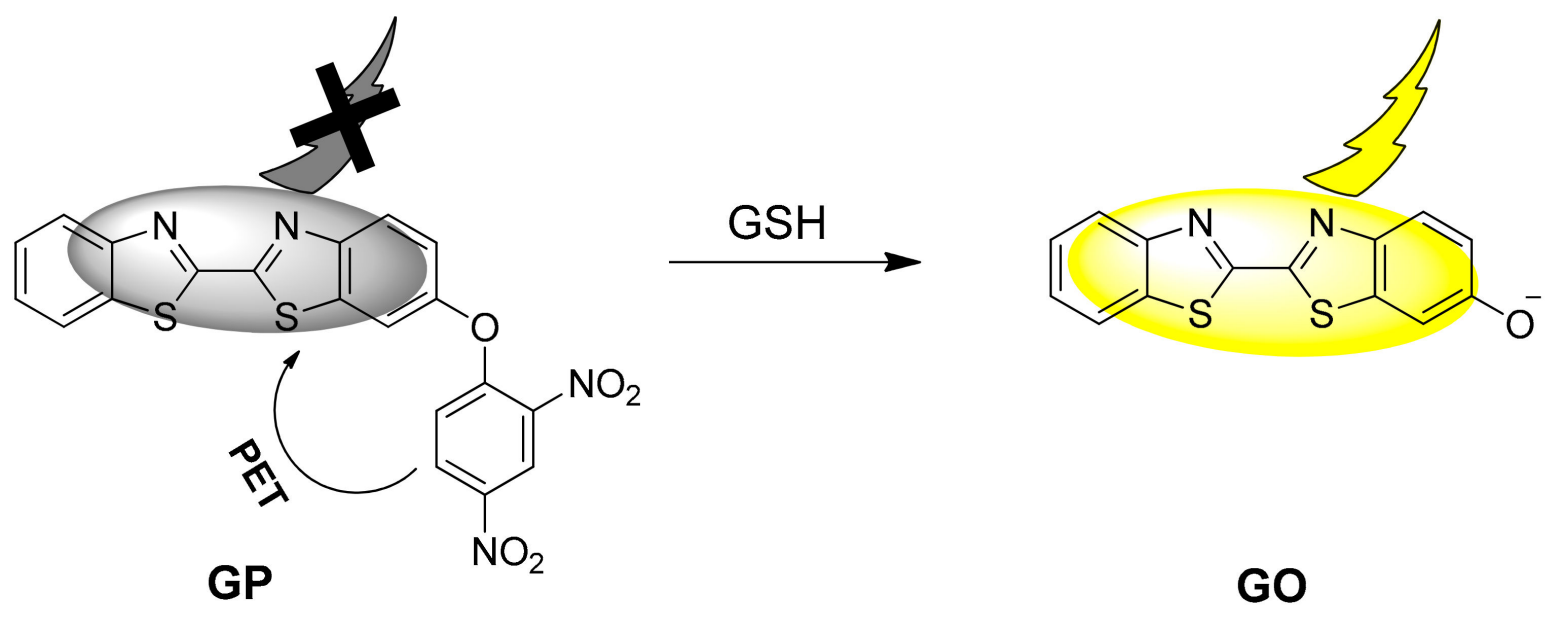
Molecular structure of probe **GP**, detection mechanism and the corresponding reaction product **GO**.

In this work, we synthesized a benzothiazole-based derivative **GP** that showed an “off–on” fluorescent change as coming across GSH in a buffer solution system (as showed in Scheme 1). Fluorescence intensity could reach the plateau after incubating in a water bath for 5 min, which presented a relatively fast responding speed. In addition, the limit of detection was 0.36 µM. The fluorescent responding product (**GO**) showed the largest Stokes shift for about 135 nm until now. We applied the probe **GP** to detect GSH in human breast cancer cells (MDA-MB-231) by a confocal laser scanning microscope and showed an obvious fluorescence enhancement to GSH compared with that in cells pretreated with a quenching agent of bio-thiols. The probe **GP** realized the fast detecting GSH and imaging GSH in living cells.

## 2. Materials and Methods

### 2.1. Materials

Regular reagents were acquired from suppliers and used without further purification. 2-Cyano-6-Hydroxybenzothiazole, 2-aminobenzenethiol, N-Ethylmaleimide, acetonitrile, 2,4-Dinitrofluorobenzeneand Sate dry dimethyl formamide (DMF) were purchased from a general reagent. K_2_CO_3_, CH_2_Cl_2_, Et_3_N, and other common reagents were purchased form Sinopharm Chemical Reagent Co., Ltd. (Shanghai, China).

### 2.2. Physical Measurements and Instrumentation

Nuclear Magnetic resonance (^1^H NMR (400 MHz) and ^13^C NMR (100 MHz)) were tested on a JEOL instrument (JEOL, Tokyo, Japan). Proton chemical shifts are reported in parts per million with tetramethylsilane (TMS) as the reference. High-resolution mass spectra (HRMS) were successfully obtained by an Agilent Technologies 6200 series TOF/6500 series Q-TOF B.06.01 (B6157) instrument (California, USA) with the negative electro-spray ionization (ESI) ionization mode. A Shimadzu RF-6000 fluorescent spectrophotometer (Kyoto, Japan) was applied to test the fluorescent spectra. CLSM images were performed on a Zeiss LSM 710 (Jena, Germany).

### 2.3. Synthesis and Characterization of Probe GP

**Scheme 2:**
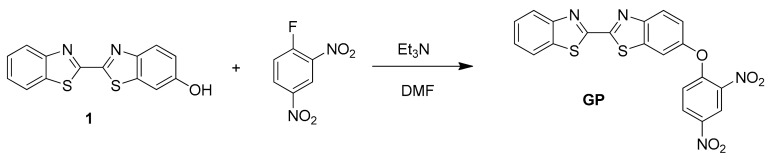
Synthesis procedure of probe **GP**.

Compound **1** was synthesized according to reported method [32]. The synthesis equation of compound **GP** was showed as Scheme 2. In addition, 50 mg of compound 1 (0.18 mmol) was dissolved in DMF. 2,4-Dinitrofluorobenzene (36 mg, 0.19 mmol) and three drops of Et_3_N were added into the system, and the mixture was stirred at room temperature for 5 h. In the reaction process, pale yellow precipitation appeared. After the reaction finished, a pale yellow product (36.5 mg, 0.81 mmol, yield = 45%) was obtained via filtration. ^1^HNMR (CDCl3, 400 MHz): δ 8.86 (d, *J* = 2.6 Hz, 1H), 8.46 (dd, *J* = 9.2 Hz, 1H), 8.25 (d, *J* = 8.9 Hz, 1H), 8.19 (d, *J* = 8.0 Hz, 1H), 8.16 (d, *J* = 8.1 Hz, 1H), 8.09 (d, *J* = 2.3 Hz, 1H), 7.60 (dt, *J* = 27.3 Hz, 7.4 Hz, 2H), 7.48 (dd, *J* = 8.9 Hz, 2.4 Hz), 7.43 (d, *J* = 9.2 Hz, 1H). ^13^CNMR: δ 162.27, 162.18, 154.69, 153.68, 153.65, 151.40, 143.14, 140.88, 137.66, 135.90, 129.88, 127.65, 127.49, 125.86, 124.20, 123.10, 122.03, 121.50, 120.36, 113.88. HRMS (ESI, *m/z*): calculated for C_20_H_10_ClN_4_O_5_S_2_ [M + Cl^−^] = 484.97866, found 484.97864.

### 2.4. Preparation of Samples and Test Solution

As the aromatic planner structure of probe **GP**, the solubility was poor in water or phosphate buffer solution (PBS), compound **GP** was dissolved in dimethylsulfoxide (DMSO) and got the stocked solution of which the concentration was 10^−3^ mol∙L^−1^. GSH, Cys, Hcy, and other amino acids were dissolved in distilled water to get a stock solution. The testing solution was obtained via diluting the stocked solution with PBS/DMSO (*v*:*v* = 7:3). The PBS concentration was 0.02 M and PH = 7.4. The mixture of probe **EP** and esterase was kept in the water bath at 37 °C for different lengths of time. In addition, the fluorescent testing was conducted with 370 nm as the excitation wavelength.

### 2.5. Calculating Method of LOD

The limit of detection (LOD) was calculated according to the following Formula:

(1) $$\mathrm{LOD}=\frac{3\sigma }{S}\;,$$

In Formula (1), σ is the standard deviation of fluorescence intensity measurements for blank sample and testing number *n* = 14; and S is the slope of liner fitting equation.

### 2.6. Confocal Laser Scanning Microscope (CLSM) Imaging

Confocal Laser Scanning Microscope (CLSM) images were conducted on a Zeiss LSM 710. The fluorescent images were collected from 530 nm to 600 nm with 405 nm laser as the excitation wavelength.

## 3. Results and Discussion

### 3.1. Preparation and Photo Physical Property

**Figure 1:**
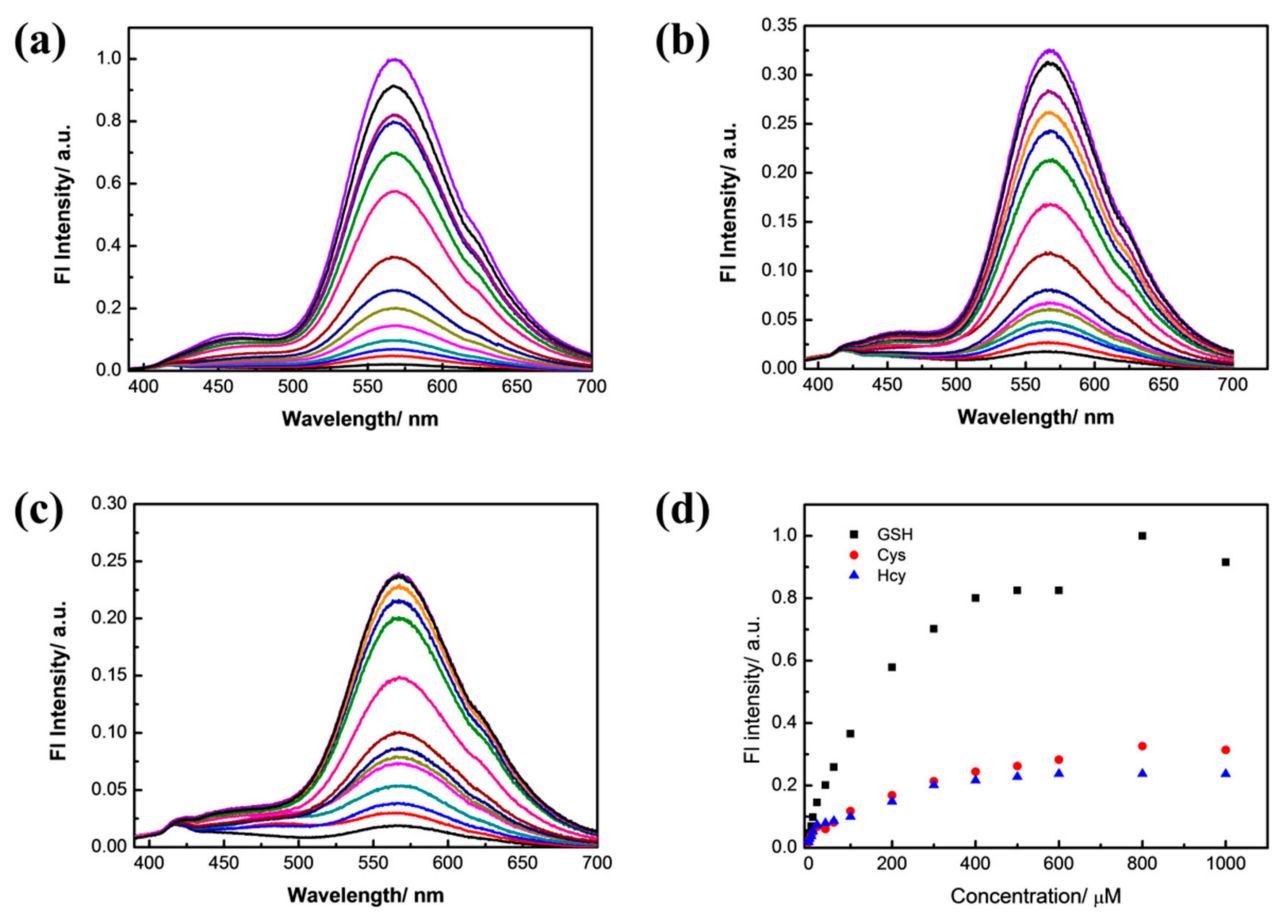
Normalized fluorescent spectra of probe **GP** (10 μM) in PBS (pH = 7.4, 0.05 M): DMSO = 7:3 after adding different concentrations (0 μM–1000 μM) of GSH (**a**), Cys (**b**), or Hcy (**c**) and incubating the mixture at 37 °C for 60 min. Fluorescence intensity at 569 nm of a mixture containing 10 μM **GP** and biothiols changes as the concentration of biothiols (GSH or Cys or Hcy) increased gradually. (**d**) Ex = 370 nm.

Probe **GP** was synthesized with compound **1** and 2,4-Dinitrofluorobenzene as the reported method [33]. With the dinitrophenyl group in molecule **GP**, photoelectron transfer (PET) [34,35,36] appeared between the dinitrophenyl group and benzothiazole fluorescent group, so no fluorescence appeared. As the nucleophilic replacement between GSH and probe **GP** in the buffer system (PBS (0.05 M): DMSO = 7:3, pH of PBS was 7.4), a strong yellow fluorescence appeared. Fluorescent emission and excitation spectra of **GO** (reaction product of **GP** solution with GSH) were tested, and it showed that the Stokes shift of **GO** was about 135 nm, which was a larger Stokes shift than those in reported references (Table S1) Fluorescence intensity of the **GP** solution showed a 50-fold increase (Figure 1a) after reacting with abundant GSH. As structures of cysteine (Cys) and homocysteine (Hcy) were similar to that of GSH, probe **GP** showed a slight increase after reacting with Cys (Figure 1b) or Hcy (Figure 1c). The fluorescence enhancement of **GP** with adding GSH, Cys, or Hcy, respectively, showed in Figure 1d, it is obvious that probe **GP** was more sensitive to detecting GSH with abundant incubating time (60 min). The limit of detection (LOD) could reflect the detection sensitivity of probe. LOD of **GP** was calculated as 0.36 µM (showed in Table S1).

**Figure 2:**
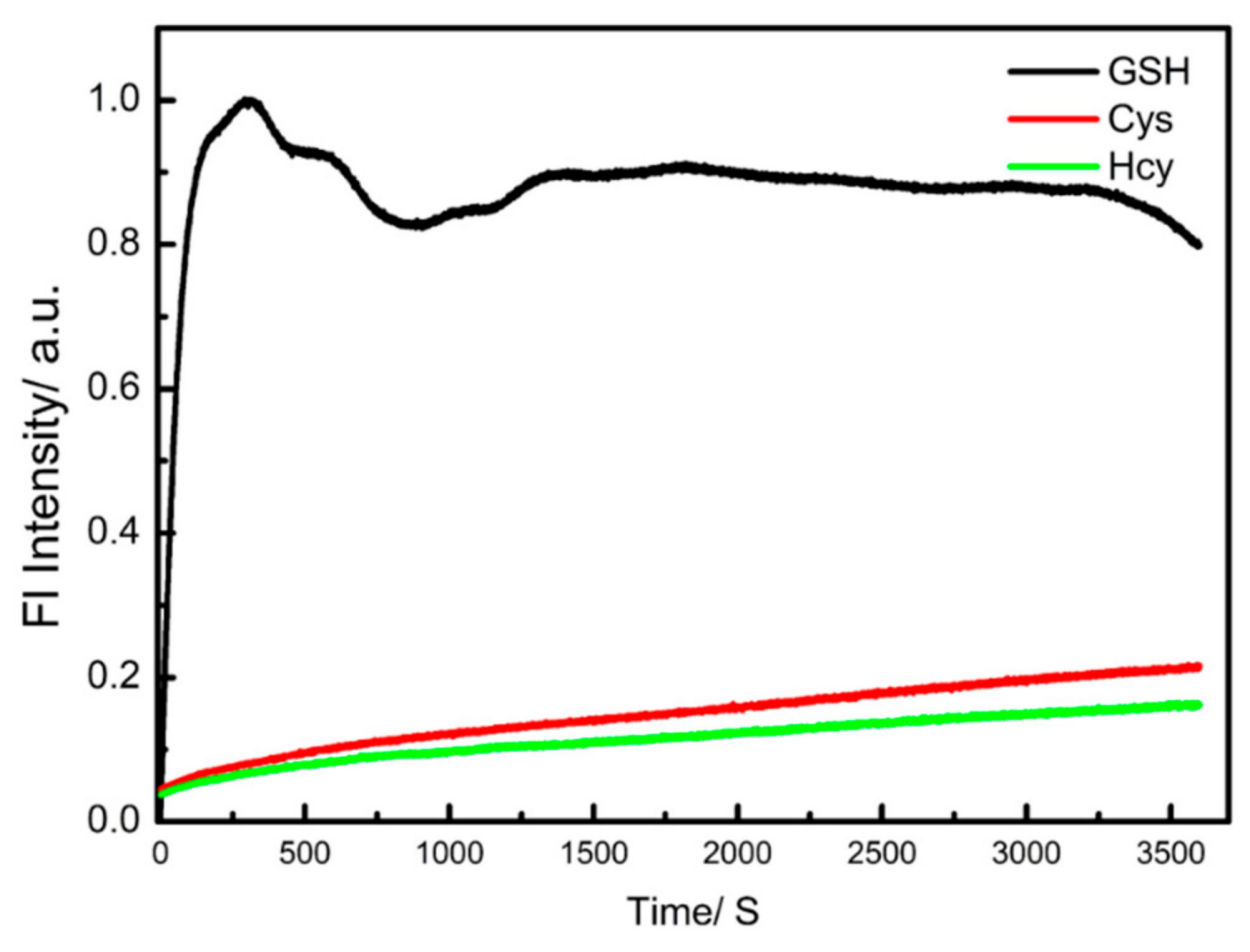
The normalized fluorescence intensity at 569 nm containing 10 μM **GP** and 1000 μM biothiols (GSH or Cys or Hcy) in PBS (pH = 7.4): DMSO (*v*:*v* = 7:3) mixed solvent change as the incubating time prolonged at 37 °C. Ex = 370 nm.

The fluorescence intensity of probe **GP** with abundant GSH, Cys, or Hcy was tested as incubated in a 37 °C water bath for different lengths of time, and the variation trend was showed in Figure 2. We found that fluorescence intensity of a mixture system containing probe **GP** and GSH increased to the platform point after incubating in a 37 °C water bath for 5 min, whereas the mixtures containing probe **GP** and Cys or Hcy showed sluggish fluorescent enhancement after incubating in a 37 °C water bath for 60 min. In this case, we can discriminate GSH from Cys/Hcy with probe **GP** after incubating for a short period of time (5 min) in a water bath.

**Figure 3:**
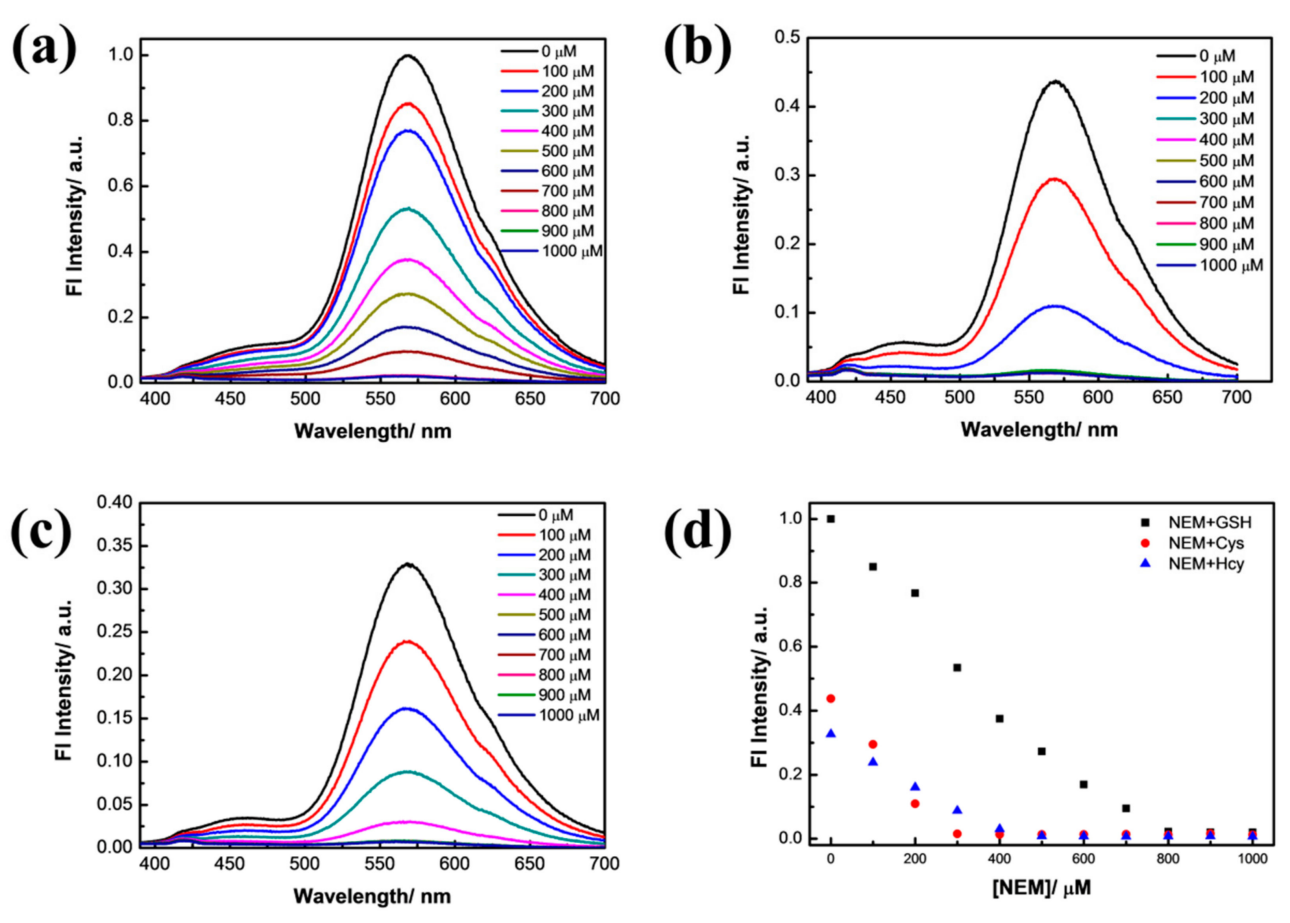
Normalized fluorescent intensity of probe **GP** (10 μM) in PBS (pH = 7.4)/DMSO mixed solvent (*v*:*v* = 7:3) containing NEM and 1000 μM biothiols ((**a**): GSH, (**b**): Cys, (**c**): Hcy) changed as the concentration of NEM increased from 0 μM to 1000 μM. The fluorescence intensity of probe **GP** at 569 nm changed as the concentration of biothiols increased (**d**). Ex = 370 nm.

To verify if the fluorescent enhancement of mixture containing **GP** and GSH was caused by nucleophilic substitution of biothiols, we try to quench the biothiols with N-Ethylmaleimide (NEM, a quenching agent of bio-thiols through thiol alkylation). Different concentrations of NEM and 1000 μM GSH or Cys or Hcy were added to the buffer solution of probe **GP**, and obvious fluorescence quenching appeared as showed in Figure 3. In addition, 800 μM NEM could quench GSH (1000 μM) absolutely, while only 400 μM NEM was needed to quench Cys (1000 μM) or Hcy (1000 μM) completely. It may be that, due to the low chemical reactivity of Cys and Hcy with probe **GP**, the required concentration of NEM was low. This result indicated that the fluorescent enhancement was indeed caused by a substitution reaction of GSH with probe **GP**.

### 3.2. Selectivity Experiment

**Figure 4:**
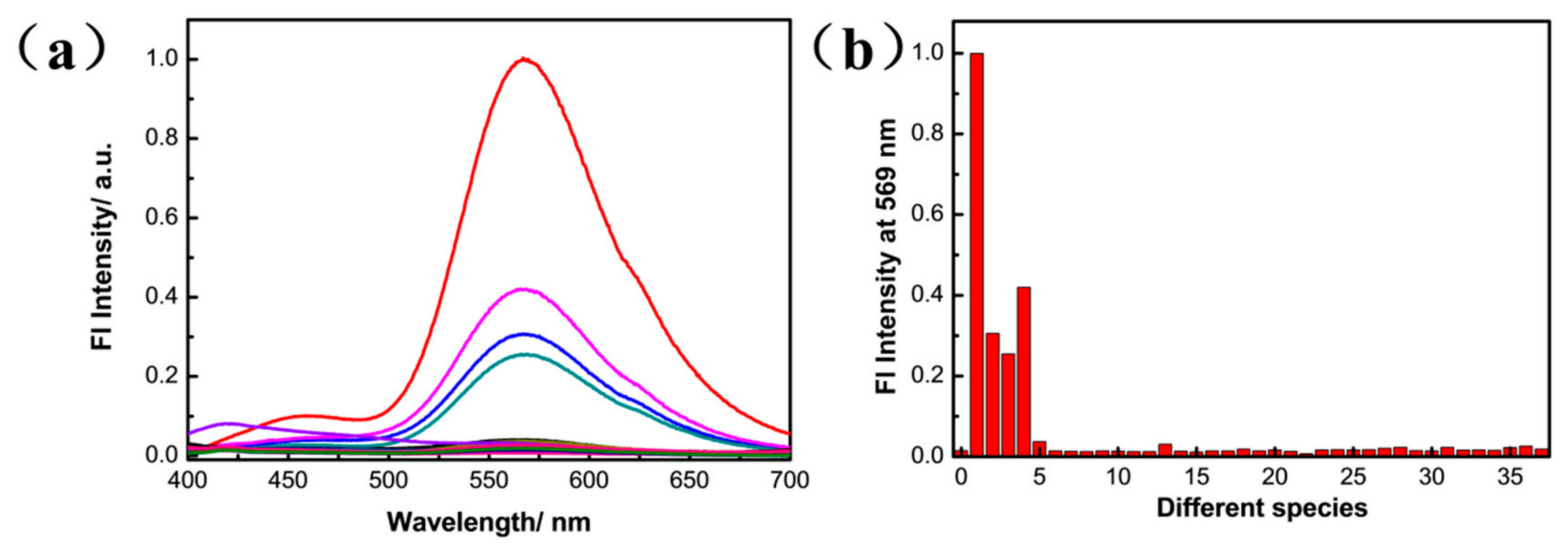
Selectivity of **GP** to other amino acids or ions. Fluorescent spectra (**a**) were conducted after adding different 1000 μM amino acids or ions into the solution of probe **GP** (10 μM). The fluorescence intensity at 569 nm was plotted as the bar graph (**b**). 0–37 represent Blank, GSH, Hcy, Cys, H_2_S, BSA, Phe, Ala, Gly, Glu, Gln, Cystine, Arg, Lys, Tyr, Leu, Pro, Trp, Ser, Thr, Asn, His, Ca^2+^, Cu^2+^, Fe^2+^, K^+^, OH^−^, Mg^2+^, Na^+^, HCO_3_^−^, Cl^−^, I^−^, Br^−^, NH_4_^+^, Sn^2+^, Zn^2+^, and PO_4_^3−^, HPO_4_^2−^. Different amino acids and ions were added into the solution of **GP** in PBS (pH = 7.4)/DMSO (7:3) and incubated in a 37 °C water bath for 60 min.

In general, fluorescent probes can be applied in cells to image target molecules. As the intracellular environment is complex and contains various proteins, amino acids, and ions, the selectivity of probe **GP** was necessary to evaluate the applicability. Different species were added into the **GP** solution and incubated mixture in a 37 °C water bath for 60 min; then, fluorescent spectra were conducted. As showed in Figure 4, Cys, Hcy, and H_2_S showed slight distractions while showing nearly no fluorescence enhancement after adding other amino acids and ions. This result indicated that probe GP showed high selectivity to other species in cells.

### 3.3. Cell Imaging Experiment and Its Application in the Analysis of Cell Healthy Status

**Figure 5:**
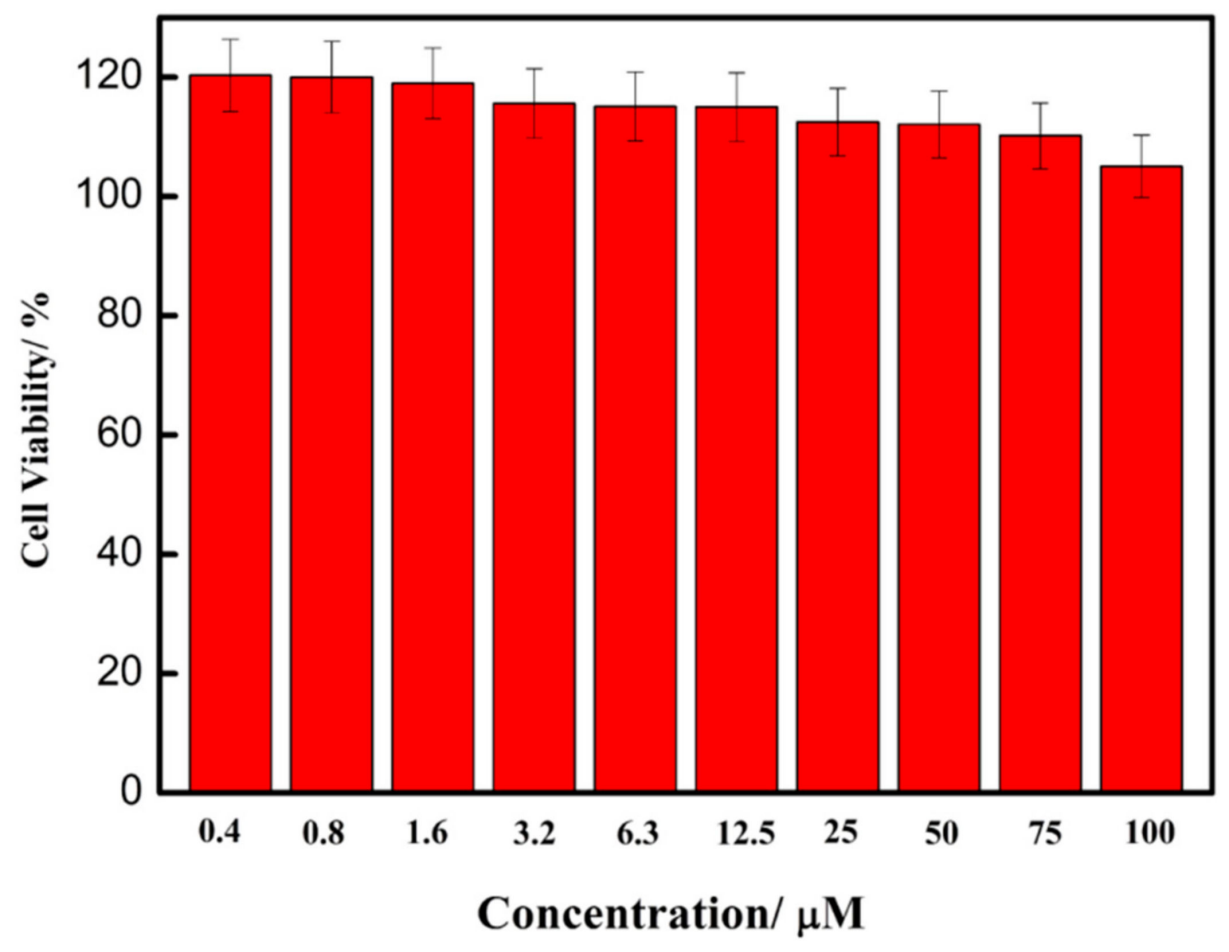
Cell toxicity of probe **GP** to MDA-MB-231 cells. Cells were incubated in cell culture fluid containing different concentrations (range from 0 μM to 100 μM) of probe **GP** at 37 °C for 24 h. The cell viability was tested via the MTT method.

We must test the toxicity of probe **GP** to MDA-MB-231 cells via the methyl thiazolyl tetrazolium (MTT) method before applying it to cell imaging. As showed in Figure 5, cell viability was still above 90% after incubating cells with different concentrations of probe **GP** (0–100 μM) for 24 h at 37 °C. This result indicated that probe **GP** was nearly nontoxic for MDA-MB-231 cells.

**Figure 6:**
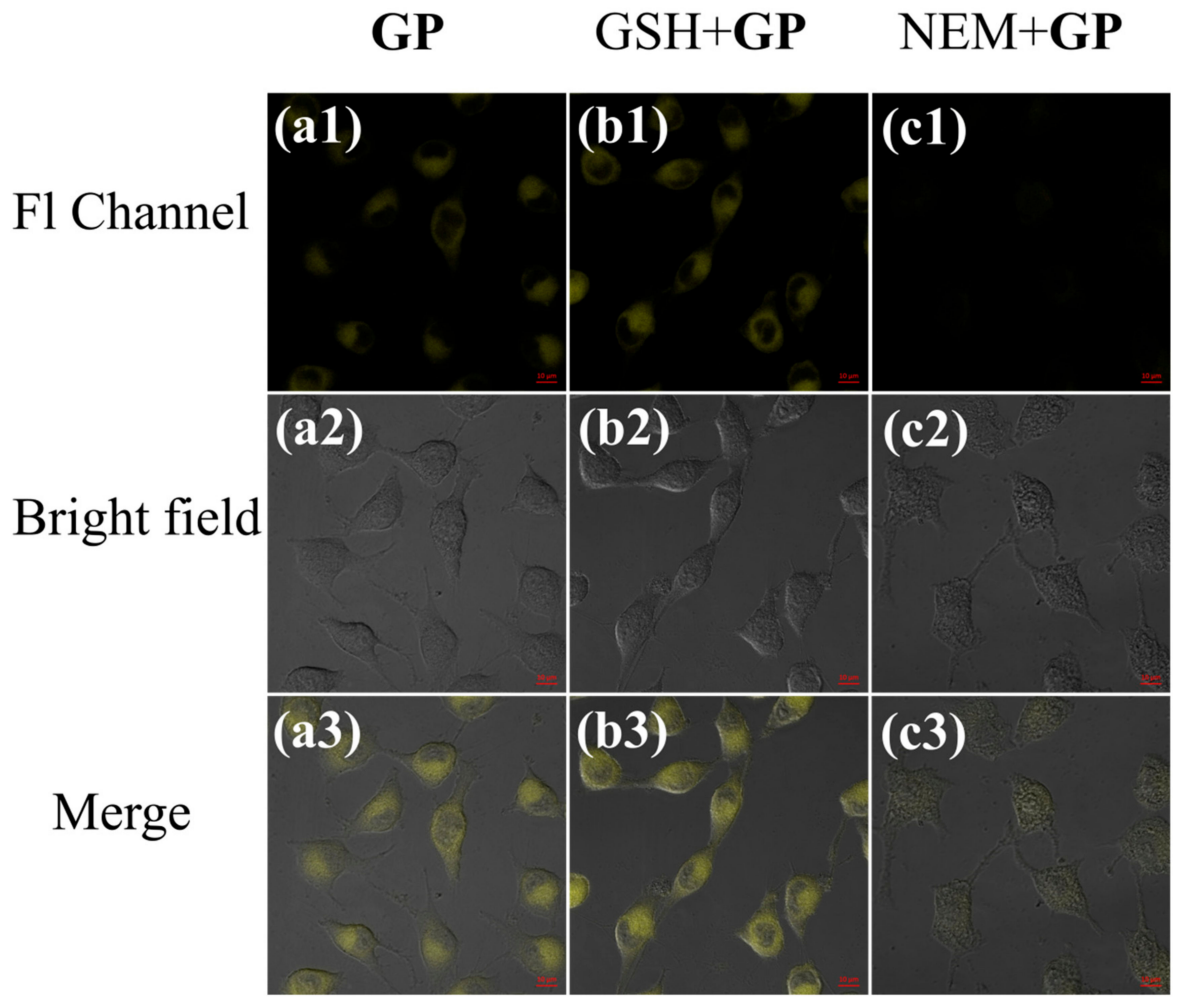
Confocal Laser Scanning Microscopy (CLSM) images of MDA-MB-231 cells incubated in PBS (pH = 7.4) containing 10 μM **GP** (**a1**–**a3**) for 30 min. MDA-MB-231 cells were pretreated with 100 μM GSH (**b1**–**b3**) or 1 mM NEM (**c1**–**c3**) for 30 min firstly and incubated in PBS containing 10 μM **GP** for 30 min. (**a1**,**b1**,**c1**): Fluorescent channel (570 nm ± 30 nm); (**a2**,**b2**,**c2**): Bright field; (**a3**,**b3**,**c3**): Overlay images of fluorescent channel and bright field. Ex = 405 nm; Scale bar = 10 μm.

Cell imaging of **GP** to cellular GSH was conducted in MDA-MB-231 cells. MDA-MB-231 cells were incubated in the mixture of PBS (pH = 7.4): DMSP = 99.1 (*v*:*v*) containing 10 μM probe **GP** for 30 min at 37 °C and then imaging was performed on a Laser Scanning Confocal Microscope (LSCM). Cells incubated with a 10 μM probe **GP** presented visible fluorescence in the yellow channel (Figure 6a1–a3). Furthermore, fluorescence in cells became more obvious (Figure 6b1–b3) when cells were pretreated with 100 μM GSH for 30 min and then incubated with 10 μM **GP** for 30 min at 37 °C. This was due to the pretreating of GSH to cells giving rise to the intracellular GSH concentration; the intracellular fluorescence induced by GSH become more obvious consequently. We evaluated the quenching effect of NEM to intracellular GSH. Cells were pretreated with 1 mM NEM for 30 min and then incubated with 10 μM probe **GP**. As showed in Figure 6c1–c3, fluorescence almost decreased to extinction and this indicated that NEM indeed quenched the intracellular GSH. These cell imaging experiment results showed that probe **GP** can image the intracellular GSH effectively.

## 4. Conclusions

In this work, we synthesized a GSH responding probe **GP** and characterized the structure via NMR and HRMS. Probe **GP** can discriminate GSH from Cys or Hcy and shows a 50-fold fluorescence increasing at 569 nm when adding GSH into a solution of **GP** in PBS/DMSO (*v*:*v* = 7:3) mixed solvent. Probe **GP** showed a short response time and fluorescence intensity reached the platform after incubating the mixture of **GP** and GSH at 37 °C for 5 min. The product of **GP** reacting with GSH possesses a large Stokes shift for 135 nm. Probe **GP** has to be applied to detect intracellular GSH and presents obvious fluorescence in MDA-MB-231 cells. This indicates that **GP** can discriminate GSH from extremely similar biothiols (Cys and Hcy) and be applied as a GSH fluorescence probe both in vitro and in living cells. As a fluorescent probe that emits near-infrared fluorescence is more appropriate to image in cells or tissues, further work is underway to shift fluorescence wavelength to near infrared region.

## Supplementary Materials

The following are available online at https://www.mdpi.com/1424-8220/19/24/5348/s1, Figure S1: Excitation (Ex) and emission (Em) spectra of GO, which is the product of GP (10 μM) reacting with GSH (1000 μM), Table S1: Limit of detection (LOD), responding time and Sticks shift of reported probes for GSH, Figure S2: ^1^HNMR of compound **GP** was conducted in d_6_-DMSO, Figure S3: ^13^CNMR of compound **GP** in d_6_-DMSO, Figure S4: HRMS of compound **GP**.
