# Supplementary material for: A GSH Fluorescent Probe with a Large Stokes Shift and Its Application in Living Cells
Source: Sensors (Basel). 2019 Dec 4;19(24):5348. doi: 10.3390/s19245348 (PMC6960701; doi:10.3390/s19245348)
Supplement: Supplementary file 1 [file sensors-19-05348-s001.pdf]

sensors

MDPI

Supplementary

A GSH Fluorescent Probe with a Large Stokes Shift and Its Application in Living Cells

Yueyuan Mao [1,2,*], Yediao Xu [1], Zhi Li [1], Yang Wang [1], Huanhuan Du [1], Lei Liu [1], Ran Ding [1] and Guodong Liu [3,*]

1 College of Chemistry and Materials Engineering, Anhui Science and Technology University, Bengbu 233030, China; 15656052160@139.com (Y.X.); lizhiusta@163.com (Z.L.); 18855036950@163.com (Y.W.); dhh122808@163.com (H.D.); liulei@feipan.org (L.L.); dingran128@126.com (R.D.)

2 State Key Laboratory of Molecular Engineering of Polymers, Fudan University, Shanghai 200433, China

3 Institute of Biomedical and Health Science, Anhui Science and Technology University, Bengbu 233030, China

* Correspondence: maoyy@ahstu.edu.cn (Y.M.); guodong.liu@ndsu.edu (G.L.)

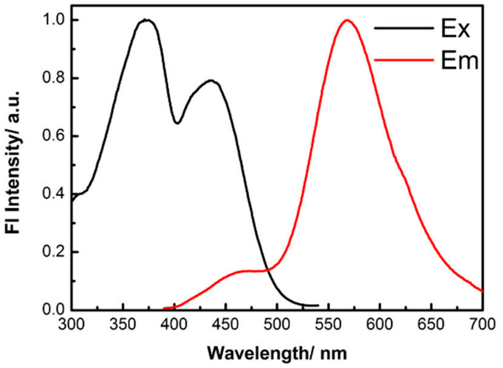

Figure S1. Excitation (Ex) and emission (Em) spectra of GO, which is the product of GP (10 μM) reacting with GSH (1000 μM). The excitation wavelength of emission spectrum is 370 nm and the settled emission wavelength of excitation spectrum is 569 nm.

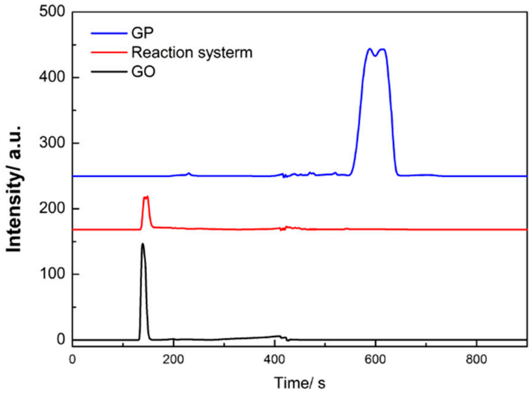

Figure S2. HPLC (High Performance Liquid Chromatography) of GP (Blue line), GO (Black line) and detecting reaction system (Red line). Acetonitrile acts as the mobile liquid phase and the velocity was 0.4 ml/min.

Table S1. Limit of detection (LOD), responding time and Sticks shift of reported probes for GSH.

Ref.	Structure	LOD	Responding Time	Type	Detection Species	Stocks Shift
This work	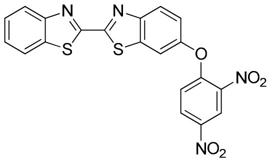	0.36 μM	5 min	on	GSH	135 nm
1[1]	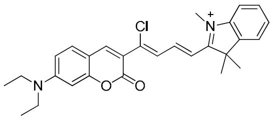	6.847μM	70 min	on	GSH	70 nm
2[2]	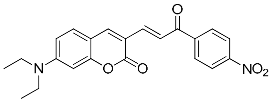	0.0065 μM	30 min	Ratiometric	GSH	100 nm
3[3]	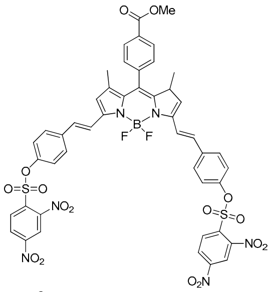	0.17μM	20 min	on	GSH	125 nm
4[4]	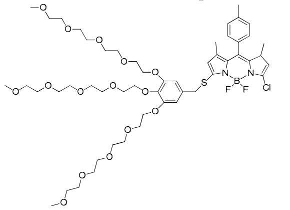	1.2μM	Not mention	on	GSH	50 nm
5[5]	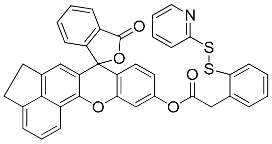	0.12μM	10 min	on	GSH/ Cys	30 nm
6[6]	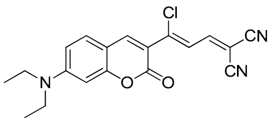	0.30μM	5 min	on	GSH	65 nm

7[7]	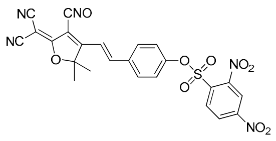	0.89μM	Not mention	on	GSH	Not mention
8[8]	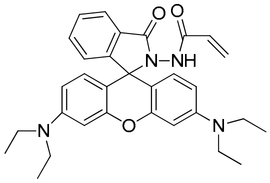	0.19μM	10 s	on	GSH	62 nm
9[9]	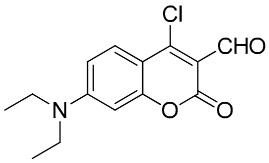	0.08μM	Not mention	Ratiometric	GSH	86 nm
10[10]	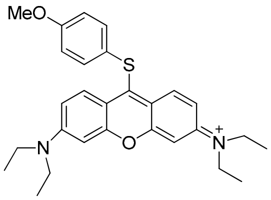	Not mention	2 min	on	GSH	40 nm
11[11]	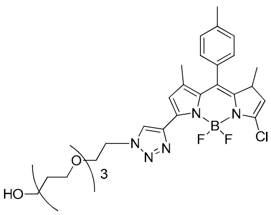	0.86 nM	10 min	Ratiometric	GSH	40 nm

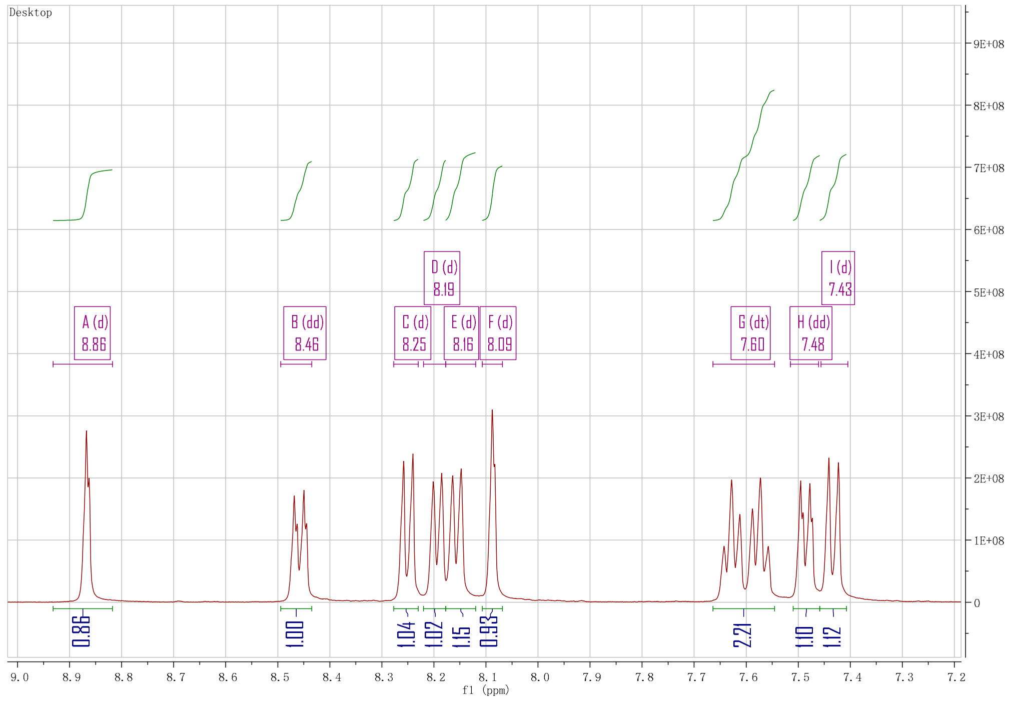

Figure S3. 1HNMR of compound GP was conducted in d6-DMSO.

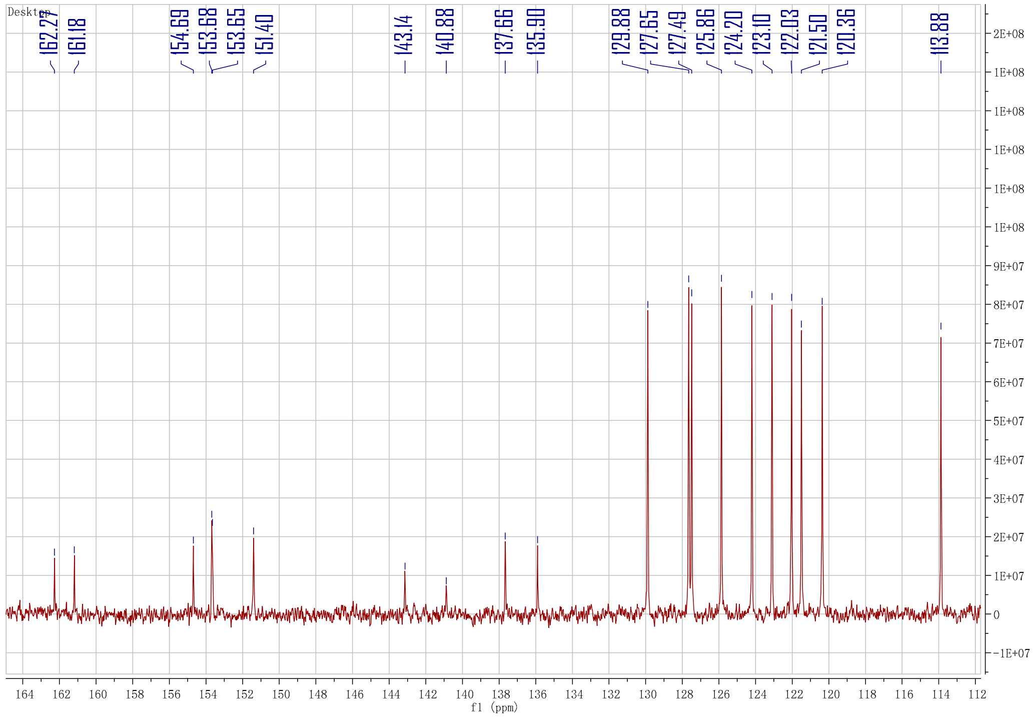

Figure S4. ^{13}CNMR of compound **GP** in d_6-DMSO.

Qualitative Analysis Report

**Data Filename**	myy-5-.d	**Sample Name**	myy-5
**Sample Type**	Sample	**Position**	Vial 21
**Instrument Name**	Instrument 1	**User Name**	
**Acq Method**	method-NEG.m	**Acquired Time**	9/25/2019 8:37:31 AM (UTC+08:00)
**IRM Calibration Status**	Success	**DA Method**	1.m
**Comment**			

**Sample Group**		**Info.**	
**Stream Name**	LC 1	**Acquisition Time (Local)**	9/25/2019 8:37:31 AM (UTC+08:00)
**Acquisition SW Version**	6200 series TOF/6500 series Q-TOF B.06.01 (B6157)	**TOF Driver Version**	6.00.01
**TOF Firmware Version**	17.643		

Spectra

**Collision Energy**	**Ionization Mode**
0	ESI

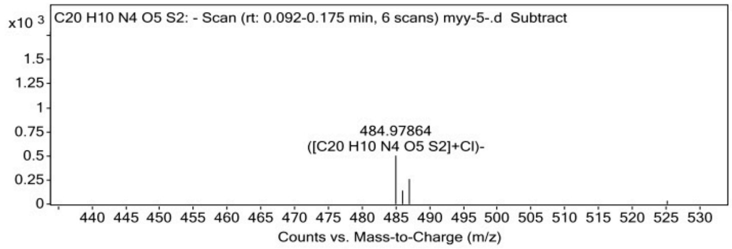

Figure S5. HRMS of compound GP.

References

1. Xiong, K.; Huo, F.; Chao, J.; Zhang, Y.; Yin, C. Colorimetric and NIR Fluorescence Probe with Multiple Binding Sites for Distinguishing Detection of Cys/Hcy and GSH in Vivo. *Anal. Chem.* **2019**, *91*, 1472–1478, doi:10.1021/acs.analchem.8b04485.
2. Xu, G.; Tang, Y.; Lin, W. Development of a Two-photon Ratiometric Fluorescent Probe for Glutathione and Its Applications in Living Cells. *Chem. Res. Chin. Univ.* **2018**, *34*, 523–527, doi:10.1007/s40242-018-8089-y.
3. Xia, X.; Qian, Y.; Shen, B. Synthesis of a BODIPY disulfonate near-infrared fluorescence-enhanced probe with high selectivity to endogenous glutathione and two-photon fluorescent turn-on through thiol-induced SNAr substitution. *J. Mater. Chem. B* **2018**, *6*, 3023–3029, doi:10.1039/c7tb03321d.
4. Liu, Y.; Niu, L.-Y.; Chen, Y.-Z.; Yang, Q.-Z. A self-assembled fluorescent nanoprobe for detection of GSH and dual-channel imaging. *J. Photochem. Photobiol. A Chem.* **2018**, *355*, 311–317, doi:10.1016/j.jphotochem.2017.08.042.
5. Hou, X.; Li, Z.; Li, B.; Liu, C.; Xu, Z. An "off-on" fluorescein-based colormetric and fluorescent probe for the detection of glutathione and cysteine over homocysteine and its application for cell imaging. *Sens. Actuators B Chem.* **2018**, *260*, 295–302, doi:10.1016/j.snb.2018.01.013.
6. Li, X.; Huo, F.; Yue, Y.; Zhang, Y.; Yin, C. A coumarin-based "0off-on" sensor for fluorescence selectivily discriminating GSH from Cys/Hcy and its bioimaging in living cells. *Sens. Actuators B Chem.* **2017**, *253*, 42–49, doi:10.1016/j.snb.2017.06.120.
7. Yang, R.; Tang, Y.; Zhu, W. Ratiometric Fluorescent Probe for the Detection of Glutathione in Living Cells. *Chem. J. Chin. Univ.-Chin.* **2016**, *37*, 643–647, doi:10.7503/cjcu20150725.
8. Huang, R.; Wang, B.-B.; Si-Tu, X.-M.; Gao, T.; Wang, F.-F.; He, H.; Fan, X.-Y.; Jiang, F.-L.; Liu, Y. A lysosome-targeted fluorescent sensor for the detection of glutathione in cells with an extremely fast response. *Chem. Commun.* **2016**, *52*, 11579–11582, doi:10.1039/c6cc06750f.
9. Dai, X.; Wang, Z.-Y.; Du, Z.-F.; Cui, J.; Miao, J.-Y.; Zhao, B.-X. A colorimetric, ratiometric and water-soluble fluorescent probe for simultaneously sensing glutathione and cysteine/homocysteine. *Anal. Chim. Acta* **2015**, *900*, 103–110, doi:10.1016/j.aca.2015.10.023.
10. Liu, J.; Sun, Y.-Q.; Zhang, H.; Huo, Y.; Shi, Y.; Guo, W. Simultaneous fluorescent imaging of Cys/Hcy and GSH from different emission channels. *Chem. Sci.* **2014**, *5*, 3183–3188, doi:10.1039/c4sc00838c.

11. Niu, L.-Y.; Guan, Y.-S.; Chen, Y.-Z.; Wu, L.-Z.; Tung, C.-H.; Yang, Q.-Z. BODIPY-Based Ratiometric Fluorescent Sensor for Highly Selective Detection of Glutathione over Cysteine and Homocysteine. *J. Am. Chem. Soc.* **2012**, *134*, 18928–18931, doi:10.1021/ja309079f.
